# Superoxide Dismutases in Immune Regulation and Infectious Diseases

**DOI:** 10.3390/antiox14070809

**Published:** 2025-06-30

**Authors:** Tong Liu, Jiajin Shang, Qijun Chen

**Affiliations:** 1 Key Laboratory of Livestock Infectious Diseases, Ministry of Education, and Key Laboratory of Ruminant Infectious Disease Prevention and Control (East), Ministry of Agriculture and Rural Affairs, College of Animal Science and Veterinary Medicine, Shenyang Agricultural University, 120 Dongling Road, Shenyang 110866, China; 2 Research Unit for Pathogenic Mechanisms of Zoonotic Parasites, Chinese Academy of Medical Sciences, 120 Dongling Road, Shenyang 110866, China

**Keywords:** superoxide dismutase, immunomodulation, PTMs, infectious diseases, parasitosis

## Abstract

Superoxide dismutases (SODs) maintain redox homeostasis through the catalytic dismutation of superoxide anions, thereby affording protection to organisms against oxidative damage. The SOD family, encompassing Cu/Zn-SOD, Mn-SOD, Fe-SOD, and Ni-SOD, exhibits structural diversity and constitutes a multilevel antioxidant defense system with discrete subcellular localizations. Beyond their antioxidant functions, SODs also function as immunomodulatory proteins, regulating the maturation, proliferation, and differentiation of immune cells. They further fulfill a crucial role in host responses to parasitic infections. The current review synthesizes and critically evaluates extant research to comprehensively delineate the molecular architecture of SODs, their intricate post-translational modification (PTM) networks, and their dual regulatory mechanisms at the interface of immunomodulation and pathological processes. This review establishes a critical framework for elucidating the biological significance of redox homeostasis maintenance.

## 1. Introduction

Reactive oxygen species (ROS), including superoxide anion radicals (O_2_^•−^), hydrogen peroxide (H_2_O_2_), hydroxyl radicals (^•^OH), and singlet oxygen (^1^O_2_), are categorized as the first line of defense in the host’s innate immune system [1]. ROS can directly assist the host in killing pathogens, particularly intracellularly parasitized pathogens, through the induction of inflammatory vesicle activation and redox regulation of immune signaling [2]. However, the oxidative stress caused by the production and accumulation of excessive ROS leads to damage to important cellular components such as proteins, lipids, and nucleic acids, which, in addition to triggering disruptions in the immune responses, can induce diabetes, vitiligo, atherosclerosis, and many other diseases [3,4]. As the primary defense against ROS damage in organisms, SODs perform a pivotal function in maintaining redox homeostasis [5].

Post-translational modifications (PTMs) of SODs are central to the regulation of SOD function. Modifications such as phosphorylation, acetylation, and nitration confer plasticity to SODs in response to stress by dynamically regulating enzyme activity, subcellular localization, and protein interaction networks [6]. The physiological significance of these modifications includes influencing enzyme activity, stability, modulating interactions with other proteins, and regulating intracellular localization of the enzyme [7]. The spatiotemporal heterogeneity of PTMs is involved not only in physiological redox homeostasis but also in the dysregulation of neurodegenerative diseases and metabolic syndromes, and its dynamic network provides a molecular basis for the development of disease markers [8,9].

SODs have been recognized as important immune modulators in various disease settings. Host-derived SOD regulates the immune response by modulating the maturation, proliferation, and differentiation of immune cells [10], whereas pathogens can evade host defenses by secreting SOD to neutralize host ROS and remodel the immune microenvironment [11]. In addition, abnormal SOD function has been closely associated with inflammatory diseases, and the mechanism involves the interactive dysregulation of oxidative stress and immune metabolic pathways [12,13].

This paper presents a systematic review of SOD immunoregulatory mechanisms, comprehensively analyzing its structural characteristics, antioxidant capacity, post-translational modifications, immune regulatory networks, and implications in disease mechanisms.

## 2. SOD Structure and Antioxidant Properties

ROS is a collective term for species derived from O_2_ that are more reactive than O_2_ itself [14]. ROS are now recognized as pivotal players in a wide array of biological processes, extending beyond their traditional portrayal as mere toxic byproducts of cellular metabolism [15]. At physiological concentrations, ROS serve as critical components, modulating inflammatory responses, cell proliferation, and apoptosis [16]. Oxidative stress resulting from the generation and accumulation of excess ROS causes damage to important cellular components such as proteins, lipids, and nucleic acids in cells [17]. This duality underscores the critical need for robust antioxidant defense mechanisms to maintain cellular homeostasis. To sustain a redox equilibrium, antioxidant defenses primarily function by inhibiting the excessive accumulation of ROS. SOD catalyzes the disproportionation of superoxide anion radicals (2O_2_^−^ + 2H^+^→H_2_O_2_ + O_2_) to counteract oxygen radicals [18]. In organisms, O_2_^•−^ is generated by the mitochondrial electron transport chain (ETC), plasma membrane-associated NADPH oxidase complex (NOX), cytoplasmic xanthine oxidase, and cytochrome p450 monooxygenase [19]. SOD converts superoxide into hydrogen peroxide and oxygen molecules. Aquaporin membrane proteins transport H_2_O_2_ [20], which is then converted to water by the activities of catalase (CAT), peroxiredoxin (PRX), and glutathione peroxidase (GPX) [21]. The importance of SOD is further highlighted by the existence of multiple isoforms, each strategically localized within different cellular compartments, suggesting specialized roles in managing superoxide levels in distinct microenvironments [22].

**Figure 1:**
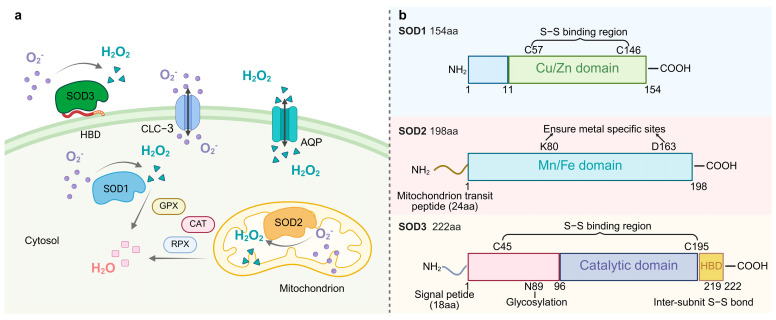
(**a**). The localization of SOD and its catalytic superoxide reaction. SOD1 (Cu/Zn-SOD) is located in the cytoplasm, SOD2 (Mn-SOD) in the mitochondria, and SOD3 (EC-SOD) in the extracellular matrix. These SODs catalyze the same reaction 2O_2_^−^ + 2H^+^→H_2_O_2_ + O_2_. Water channel proteins transport H_2_O_2_, which is then converted to water by the activities of catalase (CAT), peroxiredoxin (PRX), and glutathione peroxidase (GPX). HBD denotes heparin-binding structural domain. (**b**). The structural schematics of SOD protein family. HBD: heparin-binding domain.

The SOD family is divided into four classes based on the type of metal cofactor and phylogenetic relationship: Cu/Zn-SOD, Mn-SOD, Fe-SOD, and Ni-SOD [23]. The molecular evolutionary trajectory and subcellular localization strategies of the system under investigation have been shown to be integral components in the establishment of a spatio-temporal regulatory network of the antioxidant defense system (Figure 1).

Phylogenetic analyses indicated that Cu/Zn-SOD (SOD1) originated from a horizontal gene transfer (HGT) event in prokaryotes and differentiated into a cytoplasmic type and a plant chloroplast-type isozyme in eukaryotes through gene duplication [24]. Human SOD1, encoded by chromosome 21 [3], maintains genome stability by removing replication-associated O_2_ from the nucleus [25]. Each hSOD1 subunit is linked to Cu and Zn and contains an intramolecular disulfide bond between residues Cys57 and Cys146. This bond provides structural stability, even in unfavorable conditions, such as those found in the cytoplasm [26]. In contrast, plant chloroplast Cu/Zn-SOD (such as Arabidopsis AtCSD2) is spatially coupled to the oxygen-excreting complex (OEC) of photosystem II (PSII) [27], which specifically neutralizes ^1^O_2_ and superoxide radicals generated by photoexcitation [28]. SOD1 exists as a homodimer, with each monomer comprising a β-barrel and seven loops. These loop regions have been directly associated with SOD1 misfolding and subsequent cytotoxic aggregation [29].

Mn-SOD (SOD2) exists as a homotetramer and is localized in the mitochondria of aerobic cells [30]. The SOD2 protein has a catalytic structural domain and a mitochondrial transit peptide region. SOD2 is synthesized in cells as a precursor with a mitochondria-targeting signal peptide that enters the mitochondrial matrix via a leading sequence. The peptide is then cleaved, producing mature and enzymatically active proteins that play key roles in mitochondria [31]. Under physiological conditions, the mitochondrial ETC produces superoxide, and oxidative stress increases ROS production. Mitochondria are the main source of superoxide production in the cell, and damage to mtDNA appears to disrupt mitochondrial DNA-encoded proteins in the ETC, leading to the production of more superoxide. It has been hypothesized that Mn-SOD can impede the binding of superoxide to nitric oxide, which is produced by ETC. This theory, termed the “mitochondrial superoxide theory”, has the potential to elucidate the etiology of numerous chronic diseases, including aging and carcinogenesis [32].

Fe-SOD is considered to be the oldest form of SOD and is present in most prokaryotes [33,34]. FeSOD has been found in the archaebacterium *Methanobacterium bryantii* [35], in the eucaryote *Brassica campestris* [36], and in plant species [37], suggesting that this isoform has been present for a long period of time and has biological functions. Fe-SOD is retained by endosymbiotic gene transfer in the peroxisomes of plant chloroplasts and some postulates, and the hypoxic adaptations of its active centers are highly adapted to the micro-oxygenic microenvironment of the chloroplast-like cyst lumen [38]. The hexameric conformation of Ni-SOD, a specialized enzyme of actinomycetales and cyanobacteria, depends on the nickel ion’s “Ni-hook” coordination pattern, which is often localized in regions adjacent to the cell membrane. It traps membrane lipid peroxidation-derived O_2_^•−^ through the hydrophobic lumen [39]. Ni-SOD realizes a novel superoxide disproportionation mechanism through Ni-hook motif and hexamer assembly. Its active site switches between Ni^2+^ and Ni^3+^ and relies on electrostatic guidance with narrow channels for efficient catalysis [40]. Cys2 and Cys6 simultaneously achieve the electronic modulation of catalytic activity and stabilization of the hexamer structure by synergistically coordinating nickel centers, with Cys2 playing an irreplaceable role in maintaining the hexametric quaternary structure of the native enzyme [41].

Extracellular SOD (EC-SOD/SOD3), a specific secreted member of the Cu/Zn-SOD subfamily, is the only enzyme that mediates the disproportionation of O_2_^•−^ to H_2_O_2_ and O_2_ in the tissue extracellular matrix [3]. SOD3 has three functional domains, including a glycosylation domain, a catalytic domain, and a heparin-binding domain. The catalytic domain has 50% homology with SOD1 and has many conserved active and ionic binding sites [42]. Following secretion mediated by the N-terminal signal peptide, the enzyme is covalently anchored to the cell surface by the C-terminal heparin-binding domain, forming a stable tetrameric structure with heparan sulfate proteoglycan (HSPG) [43]. The binding of SOD3 to negatively charged extracellular matrix elements (such as heparan sulfide) and endothelial cells is important for regulating the distribution of SOD3 in the extracellular matrix [44]. This distribution feature and antioxidant properties of SOD3 are key factors in protecting extracellular matrix proteins from oxidative stress damage. SOD3 was first detected in human plasma, lymph fluid, ascites, and cerebrospinal fluid [45] and is involved in vasoprotection, placental signaling, and metabolic regulation [46]. SOD3 attenuates oxidative stress and prevents inflammation and fibrosis in various lung and vascular disease models [47], and it limits pulmonary hypertension [48]. In rats, SOD3 is expressed mainly in the epididymis [49] but also in the heart, brain, lungs, kidneys, and testes [50]. In Drosophila, SOD3 expression was highest in the nervous system, ovary, and hindgut. The expression of SOD3v2 (membrane-bound form) is significantly higher in female Drosophila than in males and may be related to the requirement for female-specific tissues such as the ovary [51]. Nematode SOD3 evolved from SOD1 adapted to the host oxidative stress environment through gene duplication and rapid differentiation and is particularly highly expressed in the female parasitic stage [52].

By virtue of the structural features of evolutionary differentiation and synergistic subcellular localization, the SOD family has established a highly efficient and hierarchical antioxidant defense system, providing a key molecular basis for the study of its function in oxidative stress-related pathological and biological adaptations.

## 3. SOD Post-Translational Modifications

### 3.1. Types of SOD Post-Translational Modifications

The activity, stability, and subcellular localization of SOD are finely regulated by a variety of PTMs, which alter the structure, activity, or interactions of the enzyme through the covalent modification of specific amino acid residues. This adaptation to the demands of cellular stress or pathology is a fundamental process that maintains cellular homeostasis.

In the field of SOD PTMs, nitration is one of the most extensively studied modifications [8]. Cu/Zn-SOD loses 90% of its enzymatic activity following ONOO^−^ modification [53]. Nitration of tryptophan (Trp) 32 may effect cytotoxicity of Cu/Zn-SOD in the familial amyotrophic lateral sclerosis mouse model [54]. The inactivation of mitochondrial Mn-SOD by peroxynitrite leads to the accumulation of O_2_^•−^ and peroxynitrite, causing oxidative and nitrosative damage to mitochondrial components and subsequently leading to mitochondrial dysfunction, apoptosis, or necrosis. Peroxynitrite has been shown to mediate Mn-SOD tyrosine (Tyr) 34 nitration and inactivation. This negative cycle may lead to catastrophic mitochondrial damage [8]. This post-translational modification in Mn-SOD may be involved in the disruption of mitochondrial redox homeostasis in diseases such as asthma, pulmonary hypertension, renal ischemia-reperfusion injury, and Parkinson’s disease [55]. Both isoforms of *Trypanosoma cruzi* (*T. cruzi*) Fe-SOD are nitrified and inactivated by ONOO^−^, thereby affecting the virulence of the parasite [56]. This finding indicates that nitration of SOD may also fulfil a distinctive biological function during parasite infection. Phosphorylation of serine (Ser), threonine (Thr), or Tyr residues of SOD is an important mechanism for energy signal perception [8]. Phosphorylation of the Ser60/Ser99 double site of SOD1 promotes its translocation from the cytoplasm to the nucleus and enhances DNA oxidative damage repair [25]. T2 phosphorylation may be an intrinsic protective mechanism for stabilizing cytoplasmic SOD1. T2 phosphorylation of T2D mutations increases the stability of the natural conformation of SOD1 and reduces the cytotoxicity of A4V-SOD1 (an amyotrophic lateral sclerosis (ALS)-associated mutation) in motor neuron-like cells [57]. Ser phosphorylation of SOD2, mediated by the CDK1/cyclin B complex, enhances its tetrameric conformation, enzymatic activity, and stability, leading to a decrease in ROS levels as well as an increase in mitochondrial function and cellular resistance to radiation-induced apoptosis [58]. The process of Ca^2+^-induced dephosphorylation of mitochondrial Mn-SOD increases enzyme activity up to twofold. This evidence suggests that Mn-SOD activity may be controlled by Ca^2+^-dependent signaling pathways to regulate the steady-state levels of superoxide or H_2_O_2_ in the matrix [59].

Acetylation of the Lys71 site inactivates SOD1 activity by disrupting the binding of SOD1 to the copper chaperone of superoxide dismutase, which in turn inhibits the formation of SOD1 homodimers [60]. Acetylation of SODK122 reduces enzyme activity by masking positively charged amino acids in the active site [61]. Acetylation of SOD2Lys68 increases mitochondrial reactive oxygen species and hypoxia inducible factor (HIF) 2α activity in tumors and promotes cellular transformation toward a hyperdifferentiated cell phenotype associated with breast cancer dissemination or metastatic recurrence [62]. Increased acetylation of Mn-SOD in uterine smooth muscle tumors leads to a decrease in its enzymatic activity, whereas restored deacetylated Mn-SOD is resistant to high concentrations of ROS [63]. The inflammatory cytokines TNF-α and AngII synergistically induce damage to class III NAD+-dependent sirtuin 3 (Sirt3) in human endothelial cells. This damage has been found to increase SOD2 acetylation and enhance mitochondrial O_2_^•−^, thereby promoting the development of hypertension [64].

Glycosylation mainly affects the functionality of secreted SOD3. Its modification pattern determines the distribution and activity of the enzyme in the extracellular matrix. SOD3 is the only glycosylated SOD that features an N-glycosylation site [65]. SOD3 carries an N-glycan chain at residue Asn89, and its extracellular distribution is regulated by glycosylation [66]. Core fucosylation is required for the secretion and enzymatic activity of SOD3, which contributes to the inhibition of cell growth in non-small lung cancer cells (NSCLC) [67]. Glycosylation in Cu/Zn-SOD may lead to enzyme fragmentation, and the loss of enzyme activity may lead to physiological problems in diabetic patients [68].

S-glutathionylation of Cu/Zn-SOD has been demonstrated to result in a decline in its enzymatic activity and to promote its aggregation. The present study hypothesizes that this process is associated with the development of familial amyotrophic lateral sclerosis (FALS) [69]. Succinylation of SOD1 at the Lys122 site has been demonstrated to inhibit the SOD1-mediated inhibition of ETC complex I, consequently resulting in increased mitochondrial respiration [6]. FeSOD from the psychrophilic eubacterium *Pseudoalteromonas haloplanktis* (*P. haloplanktis*) undergoes glutathionylation at the Cys57 site. Glutathionylation represents a pivotal antioxidant strategy for the survival of *P. haloplanktis* in cold and hyperoxic environments, whereby it protects FeSOD from oxidative inactivation, particularly against peroxynitrite, while preserving its catalytic activity [70]. Human MnSOD can be glutathionylated, but the modification does not directly lead to enzyme inactivation. Inactivation is dependent on intracellular glutathione (GSH) and thiol modifications, possibly through an indirect pathway involving multiple proteins, and the process can be reversed by dithiothreitol [71]. It was also found that recombinant rat MnSOD undergoes S-glutathionylation when expressed in *Escherichia coli*, but no enzyme activity-related studies have been reported in this context [72]. An in-depth analysis of the spatiotemporal specificity of these modifications and their dysregulation mechanisms in pathological processes will provide a theoretical basis for novel therapeutic strategies targeting the SOD pathway.

### 3.2. Biological Significance of SOD Post-Translational Modifications

PTMs remodel the biological properties of SOD by covalently modifying specific amino acid residues in several dimensions: First, they regulate enzyme activity. Nitration, glycosylation, and S-glutathionylation have been identified as the principal negative regulators of SOD activity. Second, they determine subcellular localization and compartmentalization. For example, N-glycosylation of SOD3 ensures its secretion into the extracellular matrix [73]. Third, they regulate protein stability. SUMO2/3 modification of SOD1 at Lys75 increases aggregation of SOD1 mutant proteins and enhances their stability [74]. Fourth, they integrate redox signaling networks. Sirt3, a tumor suppressor located in the mitochondria, deacetylates and activates MnSOD to scavenge ROS, thereby protecting cells from oxidative damage [75]. The PTMs of SOD exhibit significant temporal and spatial heterogeneity. Under physiological conditions, the modification levels of these proteins are tightly regulated by the kinase/modifying enzyme system to maintain redox homeostasis. In contrast, under pathological conditions, such as chronic inflammation and metabolic syndrome, oxidative stress and energy metabolism disorders lead to an imbalance in modifying enzyme activities, which triggers the dysfunction of SOD and the collapse of ROS homeostasis. The strong correlation between modification dynamics and disease progression provides a key theoretical basis for the development of early diagnostic markers and targeted intervention strategies based on PTMs.

## 4. Immunomodulatory Function of SODs

SOD plays a central regulatory role in immune cell signaling and stress management, and its function is mediated through a multilevel redox signaling network. In the innate immune response, caspase-1, a core protease of inflammatory vesicles, is responsible for the maturation of IL-1β and IL-18. In the absence of SOD1, superoxide directly inhibits caspase-1 activity through oxidative modification and reduces the release of pro-inflammatory cytokines, suggesting that SOD1 plays an important role in the initiation of inflammation by regulating caspase-1 activation [76]. Macrophages are significantly influenced by SOD. Notably, SOD promotes the polarization of macrophages from the pro-inflammatory M1 phenotype to the anti-inflammatory M2 phenotype. This shift is crucial for the transition from pathogen clearance to the resolution of inflammation and tissue repair [22]. SOD1 reduces pro-inflammatory factors such as TNF-α and IL-6 and elevates IL-10 expression by regulating the p38-MAPK/NF-κB signaling axis while inhibiting the recruitment and activation of macrophages and neutrophils and suppressing the inflammatory response [77]. SOD2 deficiency increases mtROS levels in macrophages, activates NLRP3 inflammation, induces macrophage infiltration in the lungs, and promotes the expression of inflammatory factors such as IL-1β and IL-18. These processes lead to more severe inflammation and vascular remodeling [78]. SOD3 impairs antigen presentation by specifically inhibiting the expression of maturation markers (MHCII, CD80, and CD86) in DCs [79]. SOD3 inhibits the migration and infiltration of inflammatory cells (monocytes/macrophages, leukocytes) at sites of inflammation. Furthermore, it has been shown to induce neutrophil apoptosis [80] and decreases the secretion of pro-inflammatory cytokines TNF-α and MIP-2 in macrophages. In addition, SOD3 has been demonstrated to reduce the expression of the adhesion molecules VCAM-1 and ICAM-1 in vivo [81,82].

**Figure 2:**
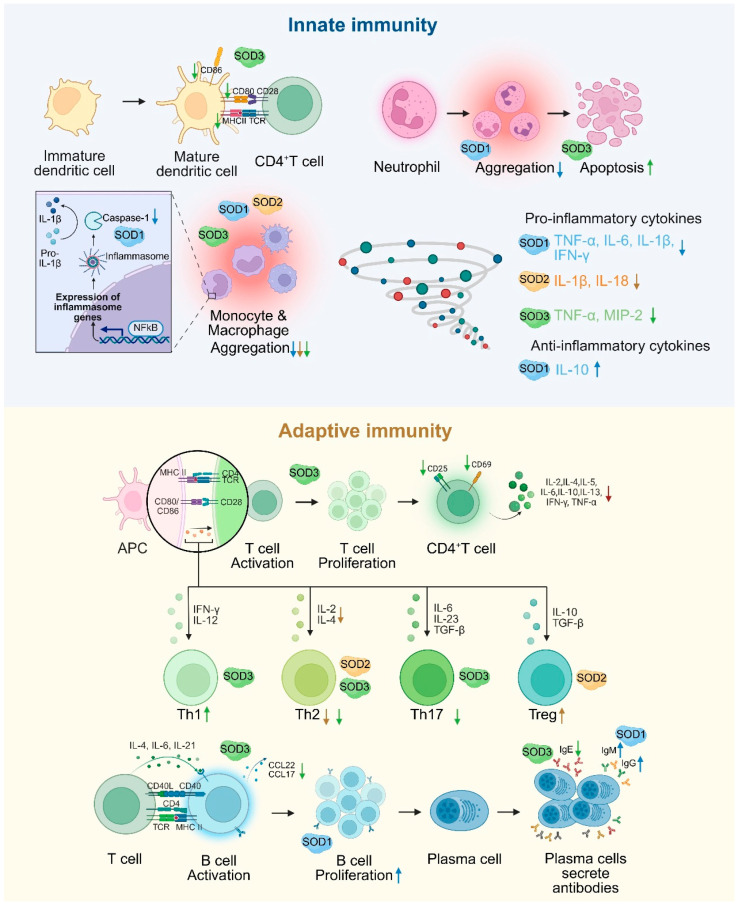
The dual regulation of SOD in innate and adaptive immunity. SOD1 regulates inflammatory vesicles, inhibits the secretion of pro-inflammatory factors, and enhances antibody production in B cells. SOD2 inhibits Th2 function and enhances Treg function by scavenging mitochondrial ROS. SOD3 restricts T/B cell activation, reduces DC maturation and inflammatory cell infiltration by inhibiting MAPK/NF-κB signaling, and balances Th1/Th2/Th17 differentiation. An upward arrow indicates promotion, and a downward arrow indicates inhibition.

In the adaptive immune response, SOD1 influences T cell activation by co-localizing with the T cell receptor (TCR), thereby ensuring the provision of oxidants necessary for the regulation of kinases and phosphatases associated with TCR signaling [83]. During the proliferative phase of B cells, recombinant human SOD1 (rhSOD1) reverses the inhibition of cell activation by scavenging superoxide. During the terminal phase of B cell differentiation, rhSOD1 promotes Ig gene transcription and enhances IgM and IgG secretion. In addition, rhSOD1 further enhances B cell proliferation and Ig secretion when combined with IL-2 or IL-4, suggesting its complementary role with cytokine signaling pathways [84]. SOD2 alleviates allergic inflammation by scavenging mitochondrial ROS and inhibiting the activation, differentiation, and function of T helper 2 cell (Th2) cells while enhancing the immunomodulatory effects of Treg cells [85]. The deficiency of Mn-SOD enhances the expression of proliferative genes in T lymphocytes as well as increased the production of cytokines and chemokines such as IL-2, IL-4, TNF-α, and CCL2 [86]. SOD3 inhibits CD4^+^ T cell activation, proliferation, and differentiation by inhibiting MAPK/NF-κB and decreasing the expression of CD25 (IL-2 receptor), CD69 (early activation marker), and CD71 (transferrin receptor), thereby limiting excessive immune response [10]. SOD3 also promotes Th1 cell differentiation but inhibits Th2 and Th17 differentiation [87], resulting in bidirectional immunomodulation. SOD3 significantly inhibits the production of IgE, the proliferation of B cells, and the production of chemokine ligands CCL22 and CCL17 in B cells by inhibiting LPS/anti-CD40-mediated activation of NF-κB, p38, and JNK. Additionally, it inhibits IgE-like switching in B cells by hindering germline gene expression at the transcriptional level [88] (Figure 2).

## 5. The Role of SOD in the Pathogenesis of Inflammatory Diseases

**Figure 3:**
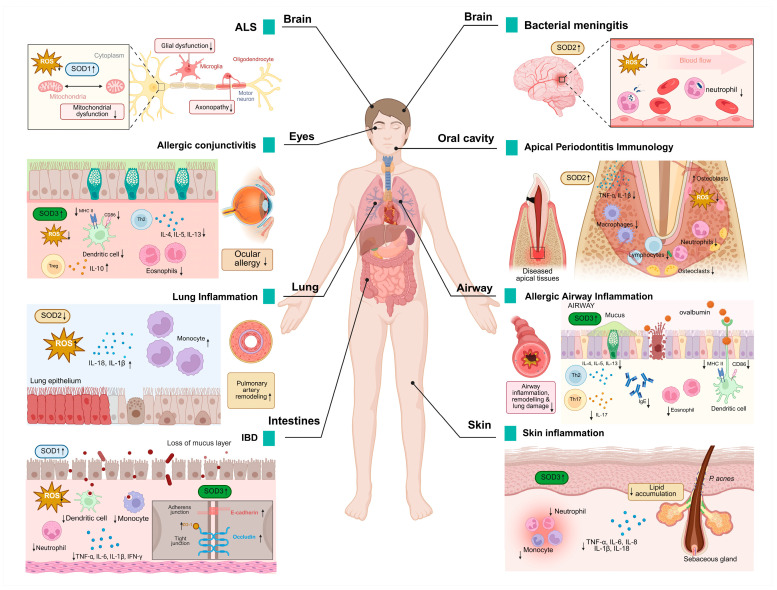
SOD expression in pathological processes of inflammatory diseases. Changes in SOD expression levels have been detected in a wide range of diseases, modulating various pathological changes such as oxidative damage, inflammatory cell infiltration, neuronal degeneration, and others. It also has a wide range of effects on different organs such as the brain, eyes, lungs, skin, and intestines. An upward arrow indicates promotion, and a downward arrow indicates inhibition.

SOD plays a “double-edged sword” role in the pathology of inflammatory diseases by regulating the dynamic balance of ROS. Abnormalities in the function of SOD not only lead directly to a vicious cycle of oxidative stress and inflammatory signaling but also contribute to the pathogenesis of numerous diseases through tissue-specific mechanisms (Figure 3).

SOD1 is the causative gene for ALS and accounts for approximately 20% of familial ALS cases [89]. SOD1 mutations drive neuroinflammation through the activation of microglia, pro-inflammatory signaling pathways (such as p38/TNF-α), and mitochondrial dysfunction, leading to motor neuron death and ALS progression [90]. SOD1 mutations trigger T-cell-mediated chronic neuroinflammation by remodeling the immune microenvironment of the central nervous system, a key driver of ALS disease progression [91].

In the context of inflammatory bowel disease (IBD), the infiltration of inflammatory cells into the inflamed gut is a pivotal mechanism that contributes to the perpetuation and escalation of inflammatory damage [92]. SOD1 inhibits DSS-induced colitis through a dual mechanism of antioxidant activity and immunomodulation. SOD1 deficiency leads to the accumulation of ROS; decreases the activity of catalytic enzymes such as glutathione peroxidase (GPx), glutathione (GSH), and catalase (CAT); exacerbates oxidative stress; and disrupts the intestinal epithelial barrier (such as decreased expression of E-cadherin), worsening colitis. Neutrophils; monocytes; pro-inflammatory CD11c^+^ macrophages and CD11b^+^CD103^−^DC; and pro-inflammatory cytokines TNF-α, IL-6, IL-1β, and IFN-γ are significantly elevated, whereas anti-inflammatory CD206^+^ macrophages and CD11b^−^CD103^+^ DC and anti-inflammatory factor IL-10 are reduced in SOD1 knockout (KO) mice [77].

In an animal model of bacterial meningitis, SOD2 expression was significantly enhanced in cerebral arterioles and small blood vessels (especially vascular smooth muscle cells and endothelial cells). This upregulation was closely associated with exposure of the cerebral vascular system to high levels of ROS [93].

Activated microglia demonstrate enhanced tolerance to oxidants (such as H_2_O_2_, xanthine/xanthine oxidase), a phenomenon that is abrogated following SOD2 knockdown. SOD2 also reduced ROS levels inside and outside of microglia, confirming the protective effect of SOD2 on cells. Conversely, SOD2 knockdown results in a significantly higher expression of inflammatory factors such as TNF-α and IL-1β and prolongs the duration of the inflammatory response, indicating that SOD2 negatively regulates inflammation. SOD2 limits excessive inflammation and prevents nerve damage by regulating the ROS-NF-κB axis [94].

The mRNA and protein levels of SOD2 were found to be significantly elevated in the gingival tissues of patients with periodontitis, especially severe cases, as verified by transcriptome analyses and clinical samples. The upregulation of SOD2 may slow down the progression of periodontal inflammation by exerting a dual effect of antioxidant and inhibition of inflammatory vesicle activity through inhibition of the NLRP3-caspase1-IL-1β axis [95].

SOD2 deficiency increases the infiltration of interstitial macrophages (F4/80^+^CD11c^−^) and monocytes (CD11b^+^Ly6C^+^) in the lungs. This increased infiltration subsequently results in the release of inflammatory mediators and the inhibition of mitochondrial superoxide radicals by scavenging the activation of the mtROS-NLRP3 inflammasome pathway, thereby reducing lung inflammation and oxidative damage [78].

SOD3 attenuates allergic conjunctivitis by modulating the Th2/Treg balance and inhibiting oxidative stress. It was shown that SOD3 inhibited Th2-type immune responses and decreased the levels of cytokines such as IL-4, IL-5, and IL-13 in draining lymph nodes. Meanwhile, SOD3 promotes regulatory T cell activity and enhances immune tolerance by upregulating IL-10. In the context of local inflammation, SOD3 inhibited the infiltration of eosinophils into conjunctival tissues and suppressed the mRNA expression of chemokines Eotaxin and RANTES. In addition, SOD3 inhibited antigen presentation and Th2 cell activation in DCs by limiting the expression of MHC class II molecules in CD11c^+^ DCs. It also scavenges ROS and blocks the inflammatory cascade mediated by the ROS-TLR4 signaling pathway [96].

SOD3 specifically inhibits dendritic cell maturation, which in turn regulates T cell activation, proliferation, and Th2/Th17 cell differentiation, thereby attenuating OVA-induced asthma symptoms (bronchoconstriction, inflammation, and airway remodeling) in mice. SOD3 intervention significantly reduces airway inflammatory cell infiltration (such as eosinophils, neutrophils), serum IgE levels, and Th2/Th17 cytokines (IL-4, IL-17, etc.) and inhibits airway collagen deposition and tissue remodeling. SOD3 controls the intensity of inflammatory signaling initiation by modulating signaling pathways such as TGF-β and EGFR and related protein interactions. This finding indicates that SOD3 may play a role in the regulation of adaptive immune responses, contributing to the inhibition of allergic asthma development [79].

SOD3 plays a protective role in colitis by scavenging ROS and modulating inflammatory responses. The study revealed that SOD3 inhibited TNF-α-induced activation of p-JNK and p-ERK and decreased the expression of pro-inflammatory factors IL-6 and IL-8 while enhancing the anti-inflammatory factor IL-10. In a DSS-induced mouse model of colitis, SOD3 or SOD3-transduced mesenchymal stem cells (SOD3-MSCs) attenuated symptoms (body weight regain, preserved colon length), repaired the intestinal epithelial barrier (upregulation of ZO-1, occludin, and E-cadherin), and reduced spleen and lymph node inflammation. In vitro experiments confirm that SOD3 protects Caco-2 cell tight junction integrity and attenuates oxidative stress and pro-inflammatory factor damage to intestinal organoids [97].

SOD3 significantly attenuated *Propionibacterium acnes* (*P. acnes*)-induced skin inflammation by inhibiting the TLR2/p38/NF-κB signaling axis and NLRP3 inflammatory vesicle activity. It was shown that SOD3 decreased the expression of pro-inflammatory factors (TNF-α, IL-1β, IL-6, IL-8) and reduced lipid accumulation and lipogenesis-related regulators (LXR-α, PPAR-γ, SREBF-1) in keratinized and sebaceous cells. In a mouse model infected with Propionibacterium acnes, the injection of SOD3 resulted in the inhibition of inflammatory cell infiltration and the reduction of skin thickening and erythema behind the ear. These findings provide evidence that SOD3 can inhibit the development of acne [98].

## 6. The Dual Role of SOD in the Pathogenesis of Parasitic Diseases

**Figure 4:**
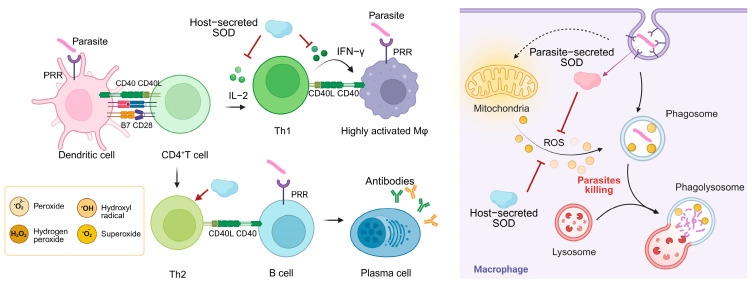
The role of different sources of SOD in host anti-parasitic immune responses and parasite immune evasion. Dendritic cells recognize the parasite and present antigenic peptides to CD4+ T cells, inducing T cell activation and differentiation. Th1 cells release IFN-γ to assist macrophage activation and generate large amounts of ROS to kill the parasite. The parasite is first surrounded by the plasma membrane of the phagocyte, forming a phagosome. The phagosome fuses with one or more intracellular lysosomes to produce a phagolysosome that releases ROS and kills the parasite. The SOD system secreted by the parasite defends against oxidative killing by the host immune system and actively intervenes in the host immune response. The SOD secreted by the host helps the parasite to achieve immune evasion.

Oxidative stress is a significant factor in the complex interplay between parasitic organisms and their hosts. Parasites have a complex network of antioxidant defense systems. For example, trypanothione is central to antioxidant defense in trypanosomes, drives enzymatic cascade reactions, and is a validated drug target. Ovothiol is enriched in the infective stage and may be associated with oxidative stress in the host environment [99]. SOD is a key antioxidant enzyme employed by many parasites to ensure their survival within the host [100]. Host immune cells (such as macrophages, neutrophils) directly attack the parasite by generating ROS, which destroy its cell membrane, proteins, and nucleic acids, and ROS are directly toxic to the parasite and cause its death. Parasites have evolved a highly efficient SOD system to resist oxidative killing by the host immune system and actively intervene in the host immune response [101] (Figure 4).

### 6.1. Parasite-Derived SOD

Oocysts are the central vehicle for *Toxoplasma gondii* (*T. gondii*) to complete its life cycle and spread to hosts [102]. SOD3 is specifically overexpressed during *T. gondii* oocyst developmental stages and acts as a key antioxidant enzyme against environmental stresses by scavenging ROS (such as UV light or disinfectants), significantly enhancing oocyst viability [103]. Its expression level was significantly higher than that of tachyzoites and bradyzoites, and it synergized with glutathione peroxidase to maintain the structural integrity of the oocysts and the infectivity of sporozoites, which is an important molecular basis for the oocysts’ environmental tolerance and propagation ability [104].

Fe-SOD is localized on the entire surface of the tachyzoites of *Neospora caninum* (*N. caninum*), allowing it to come into direct contact with host-derived superoxide anions and neutralize toxic molecules in time to avoid oxidative damage to the parasite. Fe-SOD is consistently expressed in both tachyzoite and bradyzoite developmental stages, suggesting that it plays a key protective role in both the transmission and chronic infection stages of *N. caninum* [105]. FeSODA (Fe-SOD isoform of *Leishmania donovani*) is localized to mitochondria through its N-terminal leading sequence, which directly neutralizes mitochondria-generated ROS and prevents oxidative damage [106]. The deletion of FeSODA not only directly impedes parasite proliferation by triggering G2/M-phase cell cycle arrest but also significantly reduces its survival in macrophages, suggesting that this enzyme has a key role in growth regulation and host intracellular environmental adaptation [107]. *Leishmania major*, a parasitic protozoan, expresses superoxide dismutase B1 (SODB1), which is important for parasite survival and elicits a strong immune response in the host. However, immunization with recombinant SODB1, while inducing a TH1 response, did not provide protection against infection and even appeared to exacerbate the disease in a murine model. This suggests that the immune response to parasite SODs is not always straightforward and can sometimes have unintended consequences [108].

*Plasmodium falciparum* has two types of SOD. *Pf*SOD1 is an iron-dependent SOD in the cytoplasm that is expressed throughout the erythrocytic cycle. It functions as a crucial enzyme in the host’s defense against oxidative stress [109]. *Pf*SOD2 is the second superoxide dismutase of *P. falciparum*, and its N-terminal extension carries a mitochondrial targeting sequence that specifically localizes to parasite mitochondria. This suggests that the enzyme maintains mitochondrial function by scavenging superoxide produced during mitochondrial metabolism. *Pf*SOD2 acts synergistically with *Pf*SOD1, known to work together to defend against oxidative stresses in the erythrocytic cycle [110].

*Fasciola gigantica* SOD1 (FgSOD) counteracts ROS produced by the host immune system and protects against parasite survival, migration, and reproduction in the host. The immunization of mice with recombinant FgSOD (rFgSOD) induced a dominant Th2-type immune response. The worm reduction of the rFgSOD-vaccinated group was 45.1% [111]. In in vitro experiments, the addition of exogenous SOD significantly inhibited the killing effect of immune effector cells on *F. gigantica* newly excysted juveniles (NEJs) of Schistosoma megacephalus (mortality reduced from 45% to 11%), demonstrating that SOD impairs the killing effect of the host immune response by scavenging superoxide radicals [112].

*Fasciola hepatica* regulates host immune response and immune escape by secreting SOD. FhSOD1 is continuously expressed at low levels in all developmental stages of the parasite and is released into the host environment through nonclassical secretory pathways (such as extracellular vesicles) to maintain a continuous neutralization capacity for host ROS. FhSOD3 is highly expressed in the NEJ and is guided to be exocytosed to the parasite surface through its signal peptide to break down host immune cell (such as macrophage, eosinophils) production of O_2_^•−^, reducing oxidative stress damage [11].

*Schistosoma mansoni* SOD (SmCT-SOD) expression is lowest in the larval stage and highest in the immune-tolerant adult stage, and this developmental stage-specific regulation adapts to the host microenvironment to ensure long-term adult survival. SmCT-SOD is localized at the worm–host interface (such as intestinal epithelium) and directly neutralizes host-derived ROS, protecting against oxidative killing [113]. The SmCT-SOD DNA vaccine significantly reduces worm burden by activating the Th1-type immune response (significant increase in the level of specific IgG2a antibody, increased secretion of Th1-type cytokines, such as IFN-γ and TNF-α) and specifically targeting adult *S. mansoni* worms, providing a new direction in the development of therapeutic and preventive Schistosoma haematobium vaccines [114].

SOD3 is present in the nematode excretory–secretory (ES) proteome as a secreted enzyme that acts directly on the host tissue environment to protect nematodes from localized host oxidative attack. The transcript levels and protein abundance of SOD3 were significantly higher in the parasitized female stage than in the free-living stage (upregulation could range from tens to hundreds of folds). In addition, the correlation between high expression in larval stages and its capacity to invade the host has been demonstrated [52].

As the first barrier against reactive oxygen species, FeSOD is an attractive drug target against parasitic protozoa, especially Trypanosoma and Plasmodium [115]. The parasite Fe-SODs is closely related to human mitochondrial Mn-SOD2, but there may be key differences. For example, in the substrate channel leading to the active site, lysine residues cover the active site of protozoan Fe-SOD but not mammalian SOD2. This may support the design of parasite-selective superoxide dismutase inhibitors [116].

### 6.2. Host-Derived SODs

In activated macrophages in vivo, SOD activity progressively decreased with culture time, and this decrease coincided with a decrease in cellular resistance to *T. gondii* (loss of activity after 48 h) [117]. Significantly reduced serum SOD activity in patients infected with amoebae exacerbates cell membrane damage and inflammatory responses, facilitating amoebae invasion of intestinal tissues (such as destroying the colonic mucosa, leading to hemorrhagic colitis) [118]. This reduction in SOD activity was also seen in liver tissue from cattle infected with liver flake trematodes [119].

Oxidative stress is an important clinical and pathobiochemical factor in malaria [120]. During malaria infection, SOD activity is significantly reduced in patients’ erythrocytes, leading to diminished ROS scavenging, exacerbating oxidative stress, triggering erythrocyte lipid peroxidation (such as elevated MDA) and cellular damage, and facilitating *P. falciparum* proliferation and pathologic progression [121]. SOD levels exhibited a negative correlation with parasite density, suggesting that high parasite loads may be associated with reduced SOD activity [122]. *Plasmodium berghei* ingests SOD1 from host erythrocytes in response to oxidative stress, exemplifying the evolutionary dependence of *Plasmodium* on host resources [123]. In addition, SOD3 indirectly affects the living environment of parasites by inhibiting host IL-2/IFN-γ-dependent T-cell responses, suggesting that SOD3 is a key host factor in parasite immune escape [124].

In *T. cruzi*-infected mice, Mn-SOD activity and expression were significantly reduced, leading to impaired function of the mitochondrial respiratory chain complexes I and II, decreased ATP synthesis capacity, and triggered disorders of energy metabolism. Mn-SOD deficiency directly exacerbates mitochondrial ROS accumulation and oxidative damage after *T. cruzi* infection, leading to impaired energy metabolism, myocardial inflammation, and structural damage, a key mechanism in the progression of Chagas disease [125].

The interaction between the parasite and the host SOD system constitutes a central aspect of infection pathology. Parasites, on the one hand, have been observed to resist host oxidative killing through high expression of SOD. Conversely, host SOD defects exacerbate infection damage. These mechanisms reveal that parasite SOD is not only an antioxidant enzyme but also a pleiotropic immunomodulatory molecule and that targeting SOD is required to balance host protection and pathogen inhibition, providing a new dimension for antiparasitic therapy.

## 7. Conclusions and Prospects

SOD, as a core enzyme in the antioxidant defense of organisms, plays a pivotal role in the maintenance of redox homeostasis, the modulation of immune response and disease processes by catalyzing the dismutation of superoxide anion. In this paper, we systematically summarize the molecular features of SOD, its post-translational modification network, and the duality of its immunoregulatory functions. On the one hand, host SOD maintains immune homeostasis by regulating macrophage inflammatory vesicle activation, T-cell metabolic reprogramming, and immune signaling pathways (such as NLRP3, NF-κB). On the other hand, pathogens reshape the immune microenvironment through the hijacking of SOD homologues to neutralize host oxidative killing to achieve escape. Aberrant SOD function is closely associated with inflammatory diseases, and the mechanism involves interactive dysregulation of oxidative stress and immunometabolic pathways. Superoxide dismutase plays a multifaceted and critical role in both the regulation of the immune system and the host response to a wide range of infectious diseases. Its involvement spans from modulating the function of various immune cells and influencing inflammatory processes to being a key target or defense mechanisms in parasitic infections.

While SOD holds significant therapeutic potential due to its ability to neutralize superoxide radicals, its clinical application is currently limited by challenges related to bioavailability, stability, and efficacy. Ongoing research continues to unravel the complex roles of different SOD isoforms and to develop innovative strategies to overcome these limitations. A deeper understanding of the context-dependent effects of SOD and its interactions with other biological systems is crucial for harnessing its full therapeutic potential in the fight against immune-related disorders and infectious diseases.

## Data Availability

Not applicable.
